# Re-use of Explanted DDD Pacemakers as VDD- Clinical Utility and Cost Effectiveness

**Published:** 2004-01-01

**Authors:** KKN Namboodiri, YP Sharma, HK Bali, A Grover

**Affiliations:** Department of Cardiology, PGIMER, Chandigarh, India

**Keywords:** Explanted pacemakers, VDD, DDD

## Abstract

Re-use of DDD pulse generators explanted from patients died of unrelated causes is associated with an additional cost of two transvenous leads if implanted as DDD itself, and high rate of infection according to some studies. We studied the clinical and economical aspects of reutilization of explanted DDD pacemakers programmed to VDD mode. Out of 28 patients who received VDD pacemaker during the period, October 2000- September 2001 in the Department of Cardiology, PGIMER, Chandigarh, 5 poor patients were implanted with explanted DDD pulse generators programmed to VDD mode. Each implantation was planned and carried out according to a standard protocol. The age ranged from 45 to 75 (mean-61) years. The indications for pacing were complete heart block (4) and second degree AV block (1). The clinical profile, costs and complications, if any were noted and followed up at regular intervals. The results were compared with patients who received new DDD pulse generators during this period. The additional cost for the atrial lead was not required in these patients. None of these patients had any local site infection. Compared to the two-lead system, the single lead system provided more rapid implantation and minimized complications associated with placement of an atrial lead. The explanted DDD pacemaker can be safely reused as VDD mode with same efficacy in selected patient population. This is associated with lower cost and complications compared to reimplantation as DDD itself.

## Introduction

Technology is outpacing the financial resources available for health care all over the world. Pacemakers are among the expensive devices, and when indicated properly they have no alternative. In many instances pacemakers outlive the patients and it would be a waste to dispose these pacemakers that are still in good condition having considerable battery life left without allowing others to benefit.  However the reuse of pacemakers is associated with widespread technical, medical, ethical and legal considerations.

Reuse of DDD pulse generators explanted from patients dying of unrelated causes is associated with an additional cost of two transvenous leads, if reimplanted in DDD mode itself. There are also reports of unacceptably high rates of infection associated with reuse of explanted pacemakers. We studied the clinical and economical aspects of utilization of explanted DDD pacemakers programmed to VDD mode.

## Materials and methods

During the period October 2000 to December 2001 (15 months) the patients who received pacemakers in PGIMER, Chandigarh were studied. Out of these patients, those who received generators explanted from patients who had died of unrelated causes were noted. We used explanted dual chamber pulse generators (Medtronic, Prodigy DR, model 7860, bipolar) after programming to VDD mode, with the use of new VDD lead (58-13.5-9F) in poor patients who could not afford a new pulse generator. Appropriate preprocedural evaluation was done in these patients to achieve optimal patient selection for VDD mode. The pulse generators had been explanted postmortem from patients who died of causes other than pacemaker failure after informed consent from the nearest relative. The generators were properly cleansed in antiseptic solution and sterilized with ethylene oxide and reliably tested for function and battery life. The recipient’s consent was also sought after proper explanation of the risks and benefits.

These patients were followed up after one month of implantation initially and three monthly thereafter. They were compared with those patients who received DDD pacing during the same period regarding cost, complications and procedural time. To assess the quality of life (QOL), a standard questionnaire (Table 1) was given to the patients during their last follow up visit of the study period. The questionnaire contained 7 questions, 3 related to the general QOL and 4 specific to the pacemaker related symptoms. Each question contained 4 responses, the individual score varying from 1-4 according to severity. The total score in each patient was calculated.

## Results

During the study period 136 pacemakers were implanted in same number of patients in this hospital, which included 53 dual chamber (DDD 25, VDD 28) and 83 single chamber (VVI/ R 78, AAI/R 5) pacemakers. Indications for the implantation included sick sinus syndrome (31), high-grade AV block (94), symptomatic trifascicular block (9) and cardiomyopathy (2). 21 cases involved reuse of pacemakers, which included 9 cases of reimplantation in the same patient (pacemaker infection-2, lead fracture or displacement-5, and pacemaker extrusion-2) and 12 cases of reuse of pacemakers explanted postmortem (dual chamber-8, single chamber-4).

Out of 8 dual chamber pacemaker generators explanted, 5 programmable DDD pulse generators were reimplanted in VDD mode. These 5 patients included one female and their age varied from 45 to 75 (mean 61) years (Table 2). Four of them had complete heart block while one had second-degree Mobitz-type II heart block. The battery life assessed prior to reuse varied from 43 to 124 (mean 89.4) months. The average battery voltage at implantation was 2.77 volts. A new VDD lead (58-13.5-9F), which costs about 20,000/-, was used in all. This reduced the extra amount spent on the two transvenous leads (2 x 18, 000/- = Rs 36,000/-) by Rs 16, 000/- per patient. As the VDD lead required only a single lead introducer for its insertion, an extra amount of Rs 2,000/- was also avoided making a net gain of Rs 18,000/-, i.e., 50% of the total expenditure of refurbishment per patient.

The fluoroscopy time spent for the procedure was also less compared to that used for DDD insertion. None of these 5 patients had any local site infection. The duration of postprocedural hospital stay was not prolonged in these patients (5.2 vs. 7.4 days; NS). These patients on follow up (mean follow up 19.2 months) showed normal atrial sensing and ventricular pacing. The quality of life, assessed based on a standard questionnaire did not reveal any significant difference between those received explanted pulse generator and newer one (22.2 vs. 24.4 points; NS). There was no incidence of pacemaker related tachycardia, pacemaker syndrome or lead displacement in any of these (Table 3).

## Discussion

We studied 5 patients who received explanted DDD programmable pulse generators, which were programmed to VDD mode prior to implantation. Analysis of cost effectiveness and safety in these poor and unaffordable patients revealed that these generators can be safely and effectively used at a significantly lower cost.

The rationale for the reuse of pacemakers is based on the following facts. The current lithium battery pacemakers have an expected life greater than 10 yrs. In high-risk subgroups of patients with coronary artery disease and atrioventricular block, the 3-year mortality approaches 60% [1] and thus many pacemakers would have more than 5 years of life left when the recipients die. Pringle et al reported a mortality rate of 58% within 2 years of last generator implant based on a retrospective examination of 169 consecutive pacemaker patient-deaths [2]. It should also be noted that in patients with severe cardiac diseases such as heart failure and cardiomyopathy, more sophisticated pacemakers are often implanted, and these patients do die earlier from their original disease. Thus the waste of pacemaker life is aggravated.

Mugica et al reviewed over 3,500 patients who had a 10-year follow up and reported no significant difference in the actuarial survival of those patients given explanted generator compared to those received newer one [3]. Rosengarten et al observed similar incidence of pacemaker related complications and survival among new and refurbished pacemakers in a prospective comparative study over a mean follow up period of 36 months [4]. Such experiences have repeatedly confirmed that when properly carried out, re-use of pacemakers does not pose any additional risk with considerable reduction in cost. The lack of widespread acceptance of pacemaker re-use is, therefore, not alone due to technical or medical considerations, but is related to ethical and legal problems associated with this approach.

The ethical and legal issues involve both the retrieval of a still usable pacemaker from a deceased patient and the selection of the patient to receive such a pacemaker. In countries such as Sweden, pacemakers are considered to be the property of the state and can be removed routinely without the need for consent from the families of patients who have died. In many other countries including Canada, US and India, once the pacemaker has been implanted regardless of the source of funding for this procedure, the device is considered to belong to the patient. Retrieval of such a device therefore requires the consent of the next of kin or the living will of the patient. In view of theoretical risks of reimplantation of cadaveric explants, informed consent of the recipient also should be sought, after proper explanation of possible risks involved in the re-use.

The clinical problem of reuse of pacemaker in the recipient is mainly based on the rate of infection and battery life. The risk of re-use is that an instrument might be improperly selected due to an inaccurate history of use, or improperly cleansed, tested or sterilized. Explanted pacemakers should be considered for re-use only when the reliable clinical record indicates that the instrument has had no malfunction and has an arbitrarily set minimum battery life of 5 years. After proper electronic testing, the pulse generators are to be washed under sterile conditions in distilled water and then gas sterilized with ethylene oxide for two hours at 55ºC and 60% humidity. The device is released for implantation after essential aeration for 48 hours at 55ºC in an aeration device or in the appropriate sterilizer. Mond et al described a high rate of infection with the reuse of explanted pacemakers [5]. Based on a retrospective case control study, which involved 100 patients who received re-used pacemakers, Linde et al concluded that the re-use of pacemakers could be carried out without increased risk to the patient provided a proper routine for technical control and sterilization is followed [6]. Experiences of other authors have also confirmed the short- and long-term safety of re-use of cadaveric explants [7-9].

None of our patients had local site infection. Battery life of the pulse generators was properly assessed prior to implantation by a pacing system analyzer in all. The average battery life of these 5 pulse generators at the time of implantation was 89.4 months (at 60 bpm, output 4 V, pulse width 0.4 ms and VDD mode). The re-use as VDD system resulted in avoiding the need of atrial lead placement leading to reduction in fluoroscopy time and virtual nonexistence of complications related to atrial lead, apart from reducing the cost significantly. The cost reduction by Rs 16,000/- per patient was significant considering the baseline economic status of these poor patients. There was no significant difference in the duration of hospital stay, need of antibiotics or additional cost to the patients. Proper atrial sensing, the most essential component of VDD pacing was also unaltered on follow up. Similarly, none of these patients had pacemaker syndrome, pacemaker-mediated tachycardia or atrial arrhythmias on follow up. There was no significant difference in the quality of life score between those received explanted pulse generator and newer one.

This study has some limitation in that the patient sample is small due to obvious problems mentioned. We didn’t assess the incidence of VVI pacing by Holter monitoring on follow up. However, with good sinus node function, programmed lower rate of 50 bpm and good atrial tracking noted at each follow up visit, significant VVI pacing in between is unlikely, though we cannot categorically deny such an occurrence. However, this issue is largely overwhelmed by the fact that most recipients were extremely poor and they were not in a position to afford anything more than the cost of the new electrode.

The re-use of pacemakers is not carried out routinely, as many physicians and affordable patients do not encourage it usually. The manufacturers are also understandably reluctant to participate in refurbishing used pacemakers and the pacemaker warranty disclaims liability to anyone except the first recipient. Yokoyama reported that re-use of explanted pacemaker constituted only 0.1% of all implanted pacemaker units in Japan in 1992 [10]. The corresponding figures from India are not available.

We could not find many publications on the re-use of explanted pacemakers from our country. Panja et al noted that the efficacy of re-used pacemaker was highly comparable to that of newly implanted one in terms of morbidity and mortality. Re-use in the same patient, but not reuse of cadaveric explants was associated with high rate of infection in their study [7]. Sethi et al re-used 13 explanted generators out of 42 refurbished pacemakers. There was no pulse generator failure or adverse reactions among these during a mean duration follow up of 34 months [8].  Balachander et al also reported the safety and efficacy of refurbished pacemakers in 140 implantations [9].  Hence, the pacemaker reuse needs to be encouraged in developing countries and rationale for reuse should be purely economical.

Concluding, the explanted programmable dual chamber pacemakers can be safely re-used in VDD mode with same efficacy in selected patient population, at significantly lower cost.

## Figures and Tables

**Table 1:**
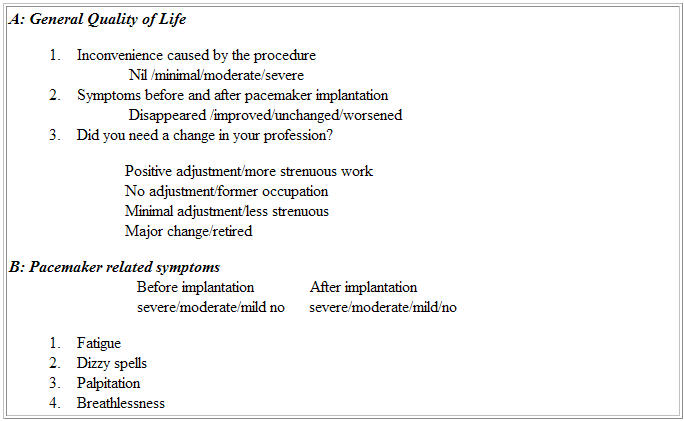
Questionnaire for Quality of life (QOL) assessment

**Table 2:**
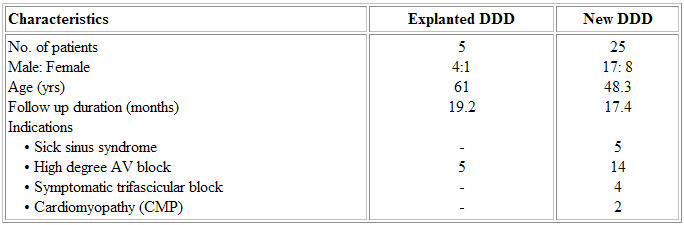
Baseline characteristics

**Table 3:**
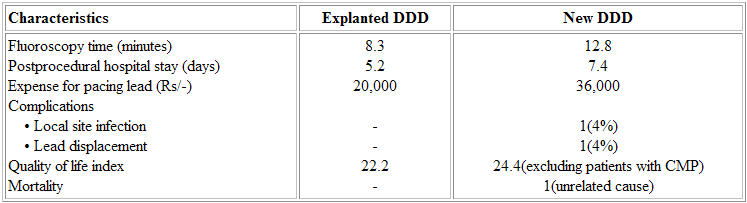
Follow-up results
